# Successful management of tracheal lobular capillary hemangioma with arterial embolization followed by electrosurgical snaring *via* flexible bronchoscopy in an 11-year-old boy: A case report and literature review

**DOI:** 10.3389/fmed.2023.1088815

**Published:** 2023-03-20

**Authors:** Xiaofen Tao, Lei Wu, Shuxian Li, Yuxin Wu, Can Lai, Enguo Chen, Zhenjie Chen, Guoping Jin, Yingshuo Wang

**Affiliations:** ^1^Department of Pulmonology, Children's Hospital, Zhejiang University School of Medicine, National Clinical Research Center for Child Health, Hangzhou, Zhejiang, China; ^2^Department of Endoscopy Center, Children's Hospital, Zhejiang University School of Medicine, National Clinical Research Center for Child Health, Hangzhou, Zhejiang, China; ^3^Zhongshan School of Medicine, Sun Yat-sen University, Guangzhou, Guangdong, China; ^4^Department of Radiology, Children's Hospital, Zhejiang University School of Medicine, National Clinical Research Center for Child Health, Hangzhou, Zhejiang, China; ^5^Department of Respiratory Medicine, Sir Run Run Shaw Hospital, Zhejiang University School of Medicine, Hangzhou, Zhejiang, China; ^6^Department of Pediatric Intensive Care Unit, Children's Hospital, Zhejiang University School of Medicine, National Clinical Research Center for Child Health, Hangzhou, Zhejiang, China

**Keywords:** lobular capillary hemangioma, tracheal, arterial embolization, electrocautery loop snaring, flexible bronchoscopy, case report

## Abstract

Lobular capillary hemangioma (LCH), previously known as pyogenic granuloma, is a benign vascular lesion commonly found within the oral and nasal cavities. However, it is rarely encountered within the trachea, especially in pediatric patients, where it manifests as hemoptysis, cough, and wheeze, and is frequently misdiagnosed as bronchitis or asthma. There is limited literature on the presentation, behavior, and management of tracheal LCH. Herein, we describe a rare case of tracheal LCH in an 11-year-old boy with a history of hemoptysis, which was successfully managed with arterial embolization followed by electrocautery loop snaring *via* flexible bronchoscopy. No complications occurred during and after the procedure. A review of the relevant literature is also provided. Our case is unique, given the therapeutic strategy utilized for pediatric tracheal LCH, and reminds physicians to be aware of tracheal LCH in the differential diagnosis for hemoptysis.

## Introduction

Primary tracheal tumors are rare, with an estimated annual incidence of 2.7 new cases per million population (1). As a benign tumor, lobular capillary hemangioma (LCH) was previously known as pyogenic granuloma and most commonly appears on the lip, nose, oral cavity, or tongue (2, 3). The etiology of this lesion remains elusive. There are many theories on pathogenesis revolving around LCH, such as a sequela of local irritation and minor trauma, neovascular response to an angiogenic stimulus with an imbalance of promoters and inhibitors, and bacterial and viral infections (3–6). As the clinical presentations are various and non-specific, LCH is often confused with true hemangiomas and granulation tissue (7, 8). Definitive diagnosis relies on histopathologic examination, which demonstrates a distinctive lobular arrangement of variably sized capillaries embedded in a fibromyxoid matrix without atypical mitoses (2, 9–11). Despite their benign nature, localized recurrence is common, and thus, surgical debulking or excision remains the mainstay of treatment if symptomatic (6, 12–14). Although there are a few studies of LCH on mucous membranes (2, 3, 6, 11, 15–17), tracheal origin for LCH is extremely rare among all primary tracheal tumors, with only four cases of children reported in the literature (9, 10, 18, 19). Thus, there is limited knowledge about the presentation, behavior, and management of tracheal LCH, which represents a diagnostic and therapeutic challenge in pediatric patients. Herein, we report a rare case of an 11-year-old boy with a tracheal LCH managed by arterial embolization, followed by electrosurgical snaring *via* flexible bronchoscopy.

## Case presentation

An 11-year-old otherwise healthy boy presented to our hospital with hemoptysis for 5 days on 20 July 2022. He had more than 10 episodes of hemoptysis per day, with 5–30 ml of bright red blood mixed with clots each time, over the preceding 5 days. Associated fatigue, dizziness, shortness of breath, chest pain, dysphagia, nasal congestion, runny nose, hematuresis, hematochezia, fever, and rigors were denied. There was no history of foreign body aspiration, trauma, hoarseness, airway intubation, or endoscopy. Initially, he was treated at a local hospital for 5 days, but he was not responding well to medical treatment with antibiotics.

**Figure 1:**
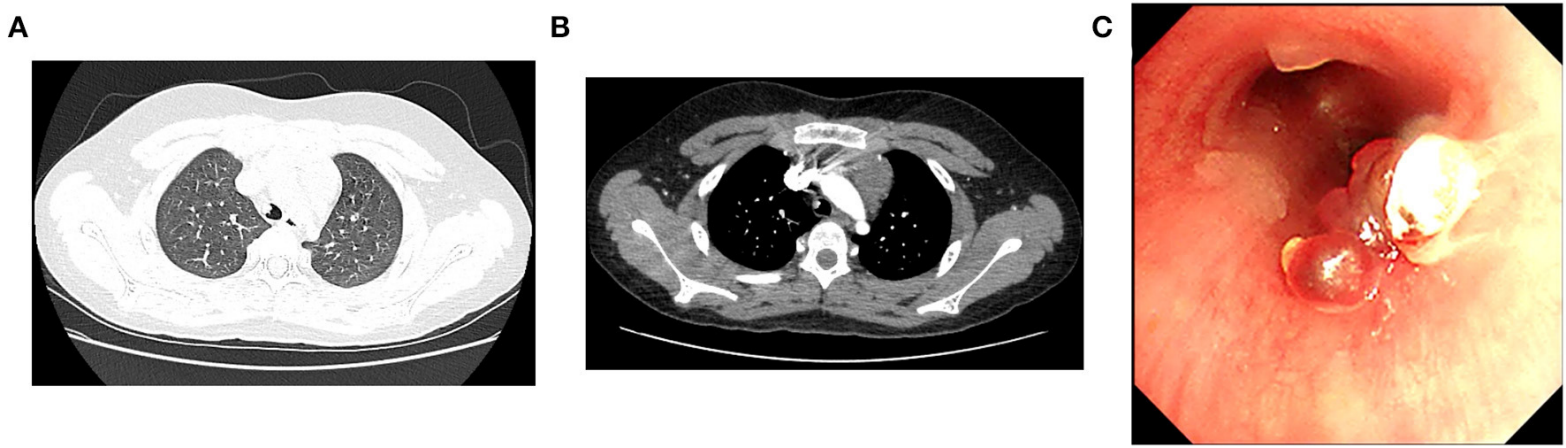
Both lung views of plain chest computed tomography (CT) scan **(A)** and mediastinal view of contrast-enhanced CT scan **(B)** demonstrate the tracheal mass. **(C)** Initial bronchoscopic view of the mass occluding the lower trachea.

On admission, he was in good condition, and the physical examination showed no abnormalities. Thoracic non-contrast-enhanced computed tomography (CT) imaging revealed a cauliflower-like lesion of 4.9^*^4.6^*^4.6 mm in size affixed to the right wall of the lower trachea (Figure 1A), with an average density of 29 Hounsfield unit (HU). Contrast-enhanced CT imaging showed an inhomogeneous enhancement in the lesion with an average density of 200 HU (Figure 1B). Flexible bronchoscopy revealed a hyperemic, broad pedunculated tracheal neoplasm, arising from the right wall of the trachea in the lower one-third and projecting into its lumen, approximately 1 cm above the main carina with blood on the surface (Figure 1C). Glomus tumor was highly suspected. To avoid potential massive tumor bleeding during the biopsy, a multidisciplinary consultation was scheduled; the boy's parents were also in favor of less invasive surgery, rather than thoracotomy. The multidisciplinary consensus was to proceed with bronchial artery embolization (BAE) for the tracheal neoplasm by interventional radiologists, and then the mass was removed with an electrocautery snare *via* flexible bronchoscopy by a pulmonologist. Surgical resection (e.g., thoracotomy) was considered as an alternative option by thoracic surgeons if procedures mentioned above failed, and the extracorporeal membrane oxygenation (ECMO) team was also on standby.

**Figure 2:**
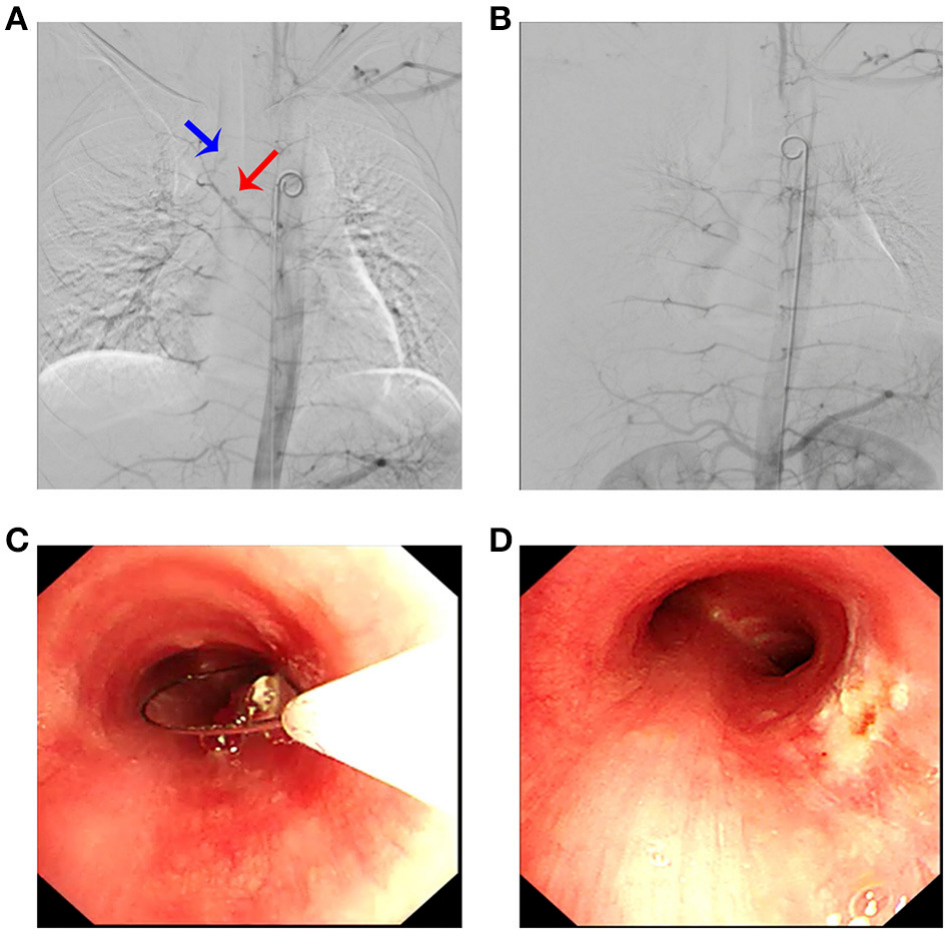
Arteriography revealed a pathological artery originating from the bronchial artery **(A)** (blue arrow: tumor; red arrow: bronchial artery branch) that was selectively embolized **(B)**. **(C)** Electrocautery loop snaring *via* flexible bronchoscopy; **(D)** posttreatment view of the same area.

In order to minimize the possibility of bleeding during lesion resection, the feeding artery was attempted to be embolized through the right common femoral artery. Digital subtraction angiography (DSA) confirmed the presence of a feeding artery (Figure 2A). Under fluoroscopic guidance, endovascular embolization was performed using a 5-Fr catheter and polyvinyl alcohol particles of 300–500 microns and 500–700 microns for distant and proximal branches, respectively. Embolization of the main bronchial artery with gelatin sponge particles was performed simultaneously. Post-embolization DSA revealed a marked reduction of the abnormal vascular blush and occlusion of the feeding artery and its branches (Figure 2B).

**Figure 3:**
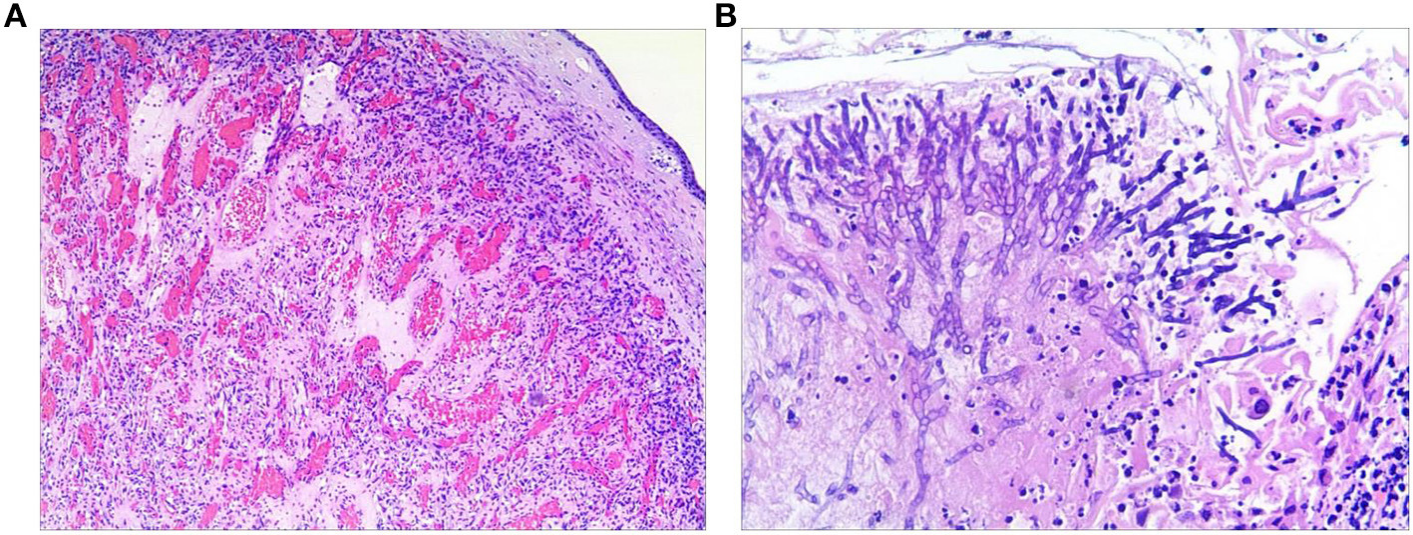
Histological examination of the resected specimen revealed capillary hemangioma **(A)** covered by the squamous epithelium and with fungal hyphae inside it **(B)**.

Then, the tumor was debulked by electrosurgical snaring with a flexible bronchoscope under general anesthesia. In detail, the electrocautery snare was used in a blend mode at 40 W to cut and coagulate the base of the mass (Figure 2C), which was resected without significant bleeding, and tracheal patency was achieved (Figure 2D). Histological examination revealed numerous capillaries arranged in a lobular pattern, separated by an edematous fibrous stroma, accompanied by mild inflammatory changes (Figure 3A) with fungal hyphae inside it (Figure 3B), which was suggestive of polypoid LCH. Immunohistochemistry revealed SMA(+), LYVB1(partial +), D2-40(-), CD34(+), WT1(+), Glut1(-), and CD31(+), further supporting the diagnosis. Although pathology suggested fungal infection, the patient did not have any typical symptoms of fungal infection throughout the course of the disease. Thus, antifungal therapy was not given during the hospitalization. The patient's postoperative recovery was uneventful, and he was discharged on the third postoperative day.

## Discussion

In the present case, the lesion was discovered while searching for causes of non-specific clinical symptoms, such as hemoptysis. It is known that primary tracheal tumors account for <2% of all upper respiratory tract tumors (20), which are usually malignant in the adult population. The most common benign tumors in the trachea include papilloma, chondroma, and fibroma, with <10% being vascular in origin (21), and LCH commonly appears on the lip, nose, oral cavity, and tongue (2, 3). These data indicate that the occurrence of LCH within the tracheobronchial tree is extremely rare and infrequently described in medical literature, especially in children. Based on CT findings, bronchoscopy was indicated and led us to the final diagnosis of tracheal LCH. To the best of our knowledge, only four pediatric cases of tracheal LCH have been reported in the literature (9, 10, 18, 19), further implying the rarity of tracheal LCH in the pediatric population. However, it should be kept in mind for the differential diagnosis of causes of hemoptysis.

Lobular capillary hemangioma (LCH), as benign vascular tumors characterized microscopically by a distinctive lobular arrangement of capillaries (2, 9, 22), was previously known as “pyogenic granulomas” or “granulomatous hemangiomas.” The traditional term “pyogenic granuloma” is inaccurate since the tumor neither contains purulent material nor is a true granuloma (11). However, the mechanism triggering the evolution of LCH is unknown, prior trauma, hormonal imbalances, infection, drug adverse effects, and genetic abnormalities associated with the nitric oxide pathway, angiogenesis and vascular injury have been proposed as predisposing factors (3–5). The possible explanation for fungal infection showed by pathology is that BAE caused local tumor tissue necrosis, and then, the necrotic material combined with the previous bleeding on the tumor surface led to the attachment of fungal hyphae to the tumor surface. Our patient had no prior history of foreign body aspiration, severe respiratory infection, signs of fungal infection, significant abnormalities, or traumatic surgery, thus, the most likely cause of our case was underlying microscopic arteriovenous malformations.

The most common clinical symptoms of tracheal LCH are cough, hemoptysis ranging from minor to massive, and expectoration. Giant masses may narrow the airway, causing dyspnea and breathlessness. The most frequent causes of hemoptysis are infectious diseases, tuberculosis, malignant tumors, cardiovascular disorders, and other inflammatory diseases. Only one of the five kids with tracheal LCH presented without hemoptysis (18) or a chronic cough. Qiu et al. summarized 12 tracheal LCH cases in the adult population and found that all of them had hemoptysis (12). Altogether, tracheal LCH should be considered a possible benign cause of hemoptysis and cough despite its rarity. Although it is a benign disease, tracheal LCH has a tremendous bleeding tendency even life-threatening. Therefore, tracheal LCH should be managed in a well-prepared manner.

The diagnosis of tracheal LCH relies on chest CT and bronchoscopy, while X-ray may show no abnormality. In detail, chest CT, especially contrast-enhanced CT, identifies the occupying lesions and reveals the nature of the mass with a plentiful blood supply, while bronchoscopy with biopsy plays a key role in achieving the final diagnosis of tracheal LCH and provides additional opportunities for therapeutic intervention. Endoscopic appearances are non-specific and sometimes may be confused with granulation tissue, carcinoid tumor, Kaposi sarcoma, angioendothelioma, paraganglioma, adenoma, angiosarcoma, intravascular angiomatosis, and carcinoma (7, 8). Hence, histopathological findings are crucial for a definite diagnosis of tracheal LCHs.

Due to the limited number of tracheal LCH cases, the preferred treatment for it has not yet been established, and therapy methods vary considerably among different studies (12, 13). Generally speaking, tracheal LCH can be treated with interventional bronchoscopy or surgical excision. Endoscopic excision, laser therapy, cryoprobe, electrocautery, brachytherapy, surgical debulking, argon plasma coagulation (APC), and cryotherapy were performed in a separate or combined way in adult tracheal LCH cases (12). The treatment experience is relatively limited in children when compared with adults because only four cases reported in the literature. The previously reported interventions for pediatric tracheal LCH include electrocauterization, cryoablation, potassium-titanyl-phosphate laser ablation, and cylindrical resection (9, 10, 18, 19), while a tracheal LCH utilizing DSA/BAE followed by electrosurgical snaring *via* flexible bronchoscopy in an 11-year-old boy was successfully removed in the present case. Collectively, although no adequate guidelines were provided and tracheal LCH is amenable to various techniques, the extent and size of the lesion, as well as patient age and comorbidities require consideration prior to any therapeutic intervention for tracheal LCH.

Although our department has rich experience in removing transbronchial tracheal tumors (23, 24), there is a substantial risk of intraoperative hemorrhage dealing with the tracheal LCH. We confront the following challenges: (1) the large size of the tumor and the rich blood supply did not allow the resection with biopsy forceps; (2) the lesion was attached to the trachea wall, and the resection with laser or cryotherapy may be associated with a high risk of perforation [6]. In the present case, DSA/BAE was employed to prevent massive intra-procedural bleeding. Of note, bronchial artery embolization ahead of tracheal LCH removal is of great importance for our case. For instance, without scheduling BAE before surgery, Dabó et al. described significant hemorrhage during tracheal LCH removal by rigid forceps *via* rigid bronchoscopy, and the instillation of cold saline and epinephrine failed to control the massive bleeding was described [20]. Inversely, in another case of BAE followed by lumpectomy under bronchoscopy, the amount of intraoperative blood loss was dramatically decreased [17]. This evidence further emphasizes that BAE was the procedure of choice in order to achieve better control of bleeding.

Meanwhile, electrocautery loop snaring was used in our case due to its ability to cut and cauterize simultaneously. The specimens obtained by electrosurgical loop snaring for diagnostic purposes are much larger and of excellent tissue quality compared with specimens extracted with forceps. Moreover, alternative surgical options are available. If a flexible bronchoscope fails to remove the tumor, a rigid bronchoscope will be employed. The endotracheal intubation should adjust the depth of endotracheal intubation if the patient loses a substantial amount of blood during the procedure, and balloon compression should be used to try to stop the bleeding. In the event that the endobronchial treatment cannot be performed as intended, the thoracic surgeon will perform a thoracotomy. If vital signs become unstable, the extracorporeal membrane oxygenation team is prepared to provide emergency care. In other words, the ECMO team and thoracic surgeons were on standby as a precautionary measure to ensure the removal of the lesion successfully. This information demonstrates the safe, successful, effective, and less invasive removal of the challenging tracheal LCH without discernible bleeding depends on close interdisciplinary cooperation and elaborate preparation.

Since the degree of tumor infiltration into the tracheal wall cannot be evaluated during bronchoscopy, individuals who have undergone bronchoscopy for tumor resection may have residual and incomplete tumor removal, and some patients may experience tumor recurrence. Future monitoring of this patient is required. In other words, it might be necessary to use bronchoscopy during the follow-up period because there is a risk of local recurrence (1).

## Conclusion

Our case is unique as it highlights the successful use of DSA/BAE followed by electrosurgical loop snaring *via* flexible bronchoscopy for the diagnosis and treatment of pediatric tracheal LCH with an abundant blood supply. Although exceeding rare, tracheal LCH should be considered a cause of recurrent hemoptysis in children. However, our experience should be validated in further large studies and further rigorous follow-up is needed for the possibility of local recurrence.

## Data availability statement

The original contributions presented in the study are included in the article/supplementary material, further inquiries can be directed to the corresponding author.

## Ethics statement

Written informed consent was obtained from the minor(s)' legal guardian/next of kin for the publication of any potentially identifiable images or data included in this article.

## Author contributions

XT and LW collected the data and drafted and edited the manuscript. SL, YWu, CL, EC, ZC, and GJ revised the manuscript. YWa supervised this study. All authors critically reviewed, revised, and approved the final manuscript and agreed to be responsible for all aspects of the study.
